# Bacterial diversity along the geothermal gradients: insights from the high-altitude Himalayan hot spring habitats of Sikkim

**DOI:** 10.1016/j.crmicr.2024.100310

**Published:** 2024-11-07

**Authors:** Santosh Kumar, Sayak Das, Namrata Jiya, Avinash Sharma, Chirantan Saha, Prayatna Sharma, Sonia Tamang, Nagendra Thakur

**Affiliations:** a Department of Microbiology, School of Life Sciences, Sikkim University, Tadong, Gangtok, Sikkim 737102, India; b Department of Life Science & Bioinformatics, Har Gobind Khurana School of Life Sciences, Assam University, Silchar, Assam 788011, India; c National Centre for Microbial Resource, National Centre for Cell Science, Pune, Maharashtra 411007, India

**Keywords:** Amplicon sequencing, Thermophiles, Mesophiles, Psychrophiles, Thermal gradient

## Abstract

Geothermal habitats present a unique opportunity to study microbial adaptation to varying temperature conditions. In such environments, distinct temperature gradients foster diverse microbial communities, each adapted to its optimal niche. However, the complex dynamics of bacterial populations in across these gradients high-altitude hot springs remain largely unexplored. We hypothesize that temperature is a primary driver of microbial diversity, and bacterial richness peaks at intermediate temperatures. To investigate this, we analysed bacterial diversity using 16S rRNA amplicon sequencing across three temperature regions: hot region of 56–65 °C (hot spring), warm region of 35–37 °C (path carrying hot spring water to the river), and cold region of 4–7 °C (river basin). Our findings showed that Bacillota was the most abundant phylum (45.51 %), followed by Pseudomonadota (32.81 %) and Actinomycetota (7.2 %). Bacillota and Chloroflexota flourished in the hot and warm regions, while Pseudomonadota thrived in cooler areas. Core microbiome analysis indicated that species richness was highest in the warm region, declining in both cold and hot regions. Interestingly, an anomaly was observed with *Staphylococcus*, which was more abundant in cases where ponds were used for bathing and recreation. In contrast, *Clostridium* was mostly found in cold regions, likely due to its viability in soil and ability to remain dormant as a spore-forming bacterium. The warm region showed the highest bacterial diversity, while richness decreased in both cold and hot regions. This highlights the temperature-dependent nature of microbial communities, with optimal diversity in moderate thermal conditions. The study offers new insights into microbial dynamics in high-altitude geothermal systems.

## Graphical abstract

**Figure:**
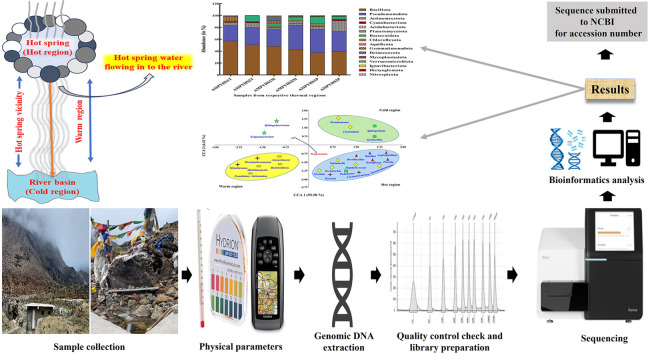

## 1 Introduction

The earth hosts various unique, unidentified and extreme niches which are inhabited by a diverse domain of bacteria, archaea and eukaryote (Singh 2017; Shu and Huang 2022; Kumar 2023). Exploring bacterial diversity in hot springs is essential for understanding microbial life in extreme environments characterized by high temperatures, variable pH levels, and unique chemical compositions (Oliverio et al. 2018; Uribe-Lorío et al. 2019; Podar et al. 2020; Mitrović et al. 2022; Shu and Huang 2022; Choudhary et al. 2024). These conditions promote the growth of extremophiles—organisms that thrive under harsh circumstances—offering insights into the limits of life and the adaptations necessary for survival (Brininger et al. 2018; Merino et al. 2019). Among them hot spring are widely studied due to its unique habitat nature and industrially importance (Cantonati et al. 2016, 2020). Hot springs are a type of geothermal phenomenon featuring a natural plumbing system, often located in extreme ecosystems (Najar et al. 2022a, 2022b). These include sulfuric acid mines, alkaline areas, extremely dry Antarctic deserts, deep snow-covered sediments, and geothermal sediments (Sharma et al. 2017, 2019; Das et al. 2020a; Jiya et al. 2024).The rich microbial communities found in hot springs provide valuable information about evolutionary processes, allowing to trace genetic and metabolic pathways and understand how organisms adapt over time. Hot springs are important in biotechnology because they host microorganisms that produce enzymes and metabolites, with various industrial applications (Urbieta et al. 2015; Yaşar Yildiz 2024). These ecosystems contribute to biogeochemical cycles, enhancing our understanding of nutrient cycling and ecosystem functionality. The unique conditions of these environments often lead to the discovery of novel microbial species, thereby expanding our understanding of biodiversity.

Numerous studies have documented the microbial community in geothermal springs globally, due to its unique habitat nature and biodiversity for research on the origin and evolution of life such as Yellowstone National Park in the USA (Blank et al. 2002; Meyer-Dombard et al. 2005; Inskeep et al. 2013; Macur et al. 2013; Payne et al. 2019; Colman et al. 2024; Felipe Benites et al. 2024), Russia (Lavrentyeva et al. 2018; Mardanov et al. 2018; Kochetkova et al. 2020, 2022; Chernitsyna et al. 2023), Croatia (Kolda et al. 2019; Marković et al. 2022; Mitrović et al. 2022; Kostešić et al. 2023; Pavić et al. 2023), Italy (Paduano et al. 2018; Pedron et al. 2019; Iacono et al. 2020), thermal springs in China (Hou et al. 2013; Tang et al. 2018; Guo et al. 2020; He et al. 2021; Ma et al. 2021; Zhang et al. 2023), hot springs in Japan (Nishiyama et al. 2013; Nishihara et al. 2018; Chen et al. 2021) and, Iceland (Aguilera et al. 2010; Reigstad et al. 2010; Mirete et al. 2011; Podar et al. 2020).

It has long been known that two of the most important variables influencing the proliferation of microbial communities in hot spring environments are temperature and pH (Loskutova et al. 2024). Thermophilic or hyperthermophilic bacteria, such as Aquificae, Deinococcus-Thermus, Thermodesulfobacteria, or Thermotogae, are enhanced when the temperature of the hot spring water rises over 75 °C (Amin et al. 2018; Tang et al. 2018; Guo et al. 2020; Kochetkova et al. 2022). In contrast, certain thermotolerant and/or mesophilic bacteria, such as Proteobacteria, Chloroflexi, and Cyanobacteria, are present at lower temperatures (<75 °C) (Lau et al. 2009; Cox et al. 2011; Guo et al. 2020). Deinococcus-Thermus, Proteobacteria, or Firmicutes predominate in alkaline hot springs (López-López et al. 2015; Lavrentyeva et al. 2018; Debnath et al. 2023), whereas thermoacidophilic communities such as Acidilobales, Sulfolobales, and Thermoplasmatales are more prevalent in acidic hot springs (Jiang and Takacs-Vesbach 2017; Mardanov et al. 2018; Guo et al. 2020). The selective benefits and distributions of hot spring microbial communities, however, are also influenced by other geochemical parameters, such as salinity, sulfide, or metal elements (Purcell et al. 2007; Briggs et al. 2014; Najar et al. 2018).

The effect of various environmental parameters such as temperature (Li et al. 2015; Mateos-Rivera et al. 2016; Chiriac et al. 2017; [Bibr bib0042][Bibr bib0061], Kumar et al., 2023a, Kumar et al., 2023b, Podar et al., 2020), pH (Rousk et al. 2010; Hossain and Sugiyama 2011; Sharp et al. 2014; Power et al. 2018; Uribe-Lorío et al. 2019), salinity (Chan et al. 2017; Benammar et al. 2024) and heavy metals (Alrumman et al. 2019; Dixit et al. 2021; Nagar et al. 2023), on bacterial community composition has been studied along the habitats. An inverse correlation between microbial diversity and temperature has been observed in various studies conducted on hot spring mud sediments (Xu et al. 2014; Chan et al. 2017) and hot springs (Podar et al. 2020). The majority of the studies point to a bell-shaped connection in which taxonomic richness decreases as habitat temperature rises and diversity peaks at moderate temperatures (Jiang et al. 2024; Negi et al. 2024). The relationship between bacterial diversity and temperature gradients that range from extremely hot (> 90 °C) to very cold (4 °C) has received relatively little attention. Moreover, distinct investigations have been carried out separately on mesophilic, thermophilic, and psychrophilic bacteria. There is not much study that have developed a thorough analysis of bacterial diversity in various temperature gradient locations. A study by Bendia et al. (2018) conducted on Sao Paulo, Deception Island, culture-dependent methods demonstrated the presence of both thermophilic and psychrophilic isolates throughout all significant temperature gradients (from 0 to 98 °C) (Bendia et al. 2018). This demonstrates that some bacteria can survive even when their metabolic activities are not permitted by the environment. Moreover, the study focuses only on the survival of bacteria in hot and cold environments only. On the other hand, Podar et al. (2020), have checked the microbial diversity in warm (38 °C) to boiling temperatures (Podar et al. 2020).

According to the Baas-Becking theory, which states that “everything is everywhere, the environment selects”, microorganisms are prevalent in the natural environment (De Wit and Bouvier 2006). However, another significant element influencing the distribution of the microbial population is geographical distance (Cho and Tiedje 2000; Papke et al. 2003; Valverde et al. 2012; Power et al. 2018).

Although it is often acknowledged that temperature plays a crucial role in microbial diversity, more investigation is needed to fully understand the intricate consequences of temperature variations in many ecological settings. Taking advantage of the opportunity, we explored the diversity of bacteria in three temperate regions: hot, warm, and cold, including samples from hot springs and nearby geothermal areas. As the hot spring water flows toward the nearest glacial river basin, it cools, creating a gradient of thermal conditions. We hypothesize that bacterial diversity will vary among these thermal regions based on their optimal temperatures, with temperature acting as the primary factor influencing microbial distribution patterns.

## 2 Materials and methods

### 2.1 Sampling site description

**Fig. 1:**
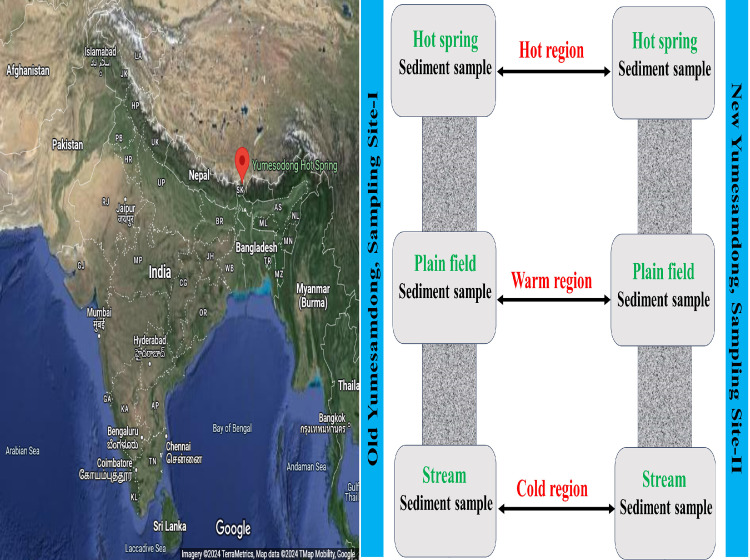
The map depicting sample location Yumesamdong hotspring, North Sikkim, India and research framework of sampling regions (hot, warm and cold).

For the present study, two separate sample sites were chosen from North Sikkim, India: Old Yusesamdong (OYS) and New Yumesamdong (NYS), as seen in Fig. 1. Both the sample locations are in close proximity to three distinct natural thermal regions, namely hot, warm, and cold. The hot spring represents the hot region (which has temperatures between 56 and 61 ± 2 °C), and as the hot spring water flows downwards it temperature decreased creating first warm region (which has temperatures 37 ± 2 °C), and finally just before hot spring water mixes with river water represent the cold region (which has temperatures between 4 and 7 ± 2 °C). The elevation of both sample locations is 4395 mamsl (meters above mean sea level) (Das et al. 2020b). The Yumesamdong hot spring is located at the base of Donkia La, approximately 25 km from Yumthang. The best thing about this spring is that it is mostly covered in snow and is in an isolated location (Kumar et al. 2023b).

### 2.2 Sample collection

Two sets of hot spring mud sediments were collected from hot, warm and cold regions of both the sampling sites (Supplementary Fig. 1). Thus, a total twelve hot spring mud sediment samples (two hot spring mud sediment samples from each site), weighing 100 gm x 12 nos. = 1200 gs collected aseptically employing sterile shovel in sterile zip-lock bags following methods as outlined by (Kumar et al. 2023b). The temperature and pH of hot spring mud sediment samples were measured using a Brannan thermometer and a Hi-media portable meter at the time of sampling as stated in Table 1. Using GPSMAP 78S (Garmin, India), the coordinates of the sampling's geographic location and elevation were determined as indicated in Table 2. Following collection, all of the samples was transported in an insulated chiller ice box filled with ice pack to the laboratory for further analysis.

**Table 1:** The physical attributes of the corresponding thermal regions.

Sampling sites		Temperature	pH
Hot region	Site-I Old Yumesamdong	56 °C	8.60
Warm region		37 °C	9.16
Cold region		7 °C	9.41
Hot region	Site-II New Yumesamdong	65 °C	8.25
Warm region		37 °C	8.90
Cold region		4 °C	9.00

**Table 2:** The geographical location and elevation of the sampling sites.

Sampling sites		Latitude	Longitude	Elevation
Site-I	Hot region	27.91770	88.69421	4395 mamsl*
	Warm region	27.91747	88.69417	
	Cold region	27.91722	88.69419	
Site-II	Hot region	27.91802	88.69401	
	Warm region	27.91791	88.69399	
	Cold region	27.91780	88.69396	

mamsl* = Meters above mean sea level.

## 3 Genomic dna isolation and purification

Genomic DNA was extracted by using DNA isolation Kit (DNeasy PowerSoil Pro Kit, Qiagen, Germany (Catalog No No 47,014) as per the given protocol by manufacturer. Extracted DNA quality is checked with the help of Nano-Drop 1000 UV–VIS Spectrophotometer (Thermo Scientific).

## 4 Sequencing and bioinformatics analysis

The two primers were used to amplify the 16S rRNA genes for V3 and V4 regions 16S rRNA- 515F and 806R (Caporaso et al. 2011). The Nextera XT Index Kit (Illumina Inc.) was used to create the amplicon libraries as described in the Illumina 16S metagenomic sequence library preparation technique (Klindworth et al. 2013). AMPure XP beads were used to purify the amplicon library. The amplified library was examined using Agilent Tapestation 4200, and the concentration was determined using a Qubit 4.0 fluorometer. 600 uL of the 8pM library was transferred into the MiSeq cartridge for cluster creation and sequencing. The method of paired-end sequencing was applied on the Illumina Miseq platform. After the sequencing, the barcode and adaptor sequences were removed from the high-quality 16S amplicon sequence reads by trimming (Faircloth et al. 2014).

Pre-processing for de-replication, singleton removal, OTU clustering, and chimera filtering with SolexaQA was performed on the adapter trimmed sequence. Sequences with a phred score of <20, ambiguous bases with mismatched primers, and short read lengths of 100 bp were eliminated. UPARSE OTU clustering and QIIME were used to annotate and standardize the operational taxonomic unit (OTU) at a 97 % similarity level (Wang et al. 2016). Both a built-in script and METAGEN aid were utilized for standardization. Using the Green Genes database (https://greengenes.lbl.gov) and SILVA, the taxonomic classification and alignment of the generated representative OTU were performed. A classification of the readings at several taxonomic levels, including kingdom, phylum, class, order, family, genus, and species, is the outcome of this workflow. Sequences without a homologous pair were labelled as unidentified.

Then the sequences were submitted to the NCBI database (https://dataview.ncbi.nlm.nih.gov/object/) and the accession numbers were obtained, as presented in Supplementary Table 4. The alpha diversity indices were calculated using EstimateS and PAST software (Hammer and Harper 2001). Alpha diversity denotes the species’ variedness and Chao value depicts the species richness among the environmental samples (Chao et al. 2006). Regression plot and corelation, *t*-test ANOVA were performed by Graph Pad Prism (version 10.2.3) software (Motulsky and Christopoulos 2004). The R software with the Bray Curtis Dissimilarity matrix (package: ggplot, function: heatmap.2) was used to create and analyse the heat map based on genus level diversity. To precisely assess the bacterial diversity pattern within each thermal regions, all the duplicates’ samples were polled collectively and then the mean were analysed. The AMPYHS1T and AMPYHS2T (for the hot regions site-I & site-II), AMPYHS1M and AMPYHS2M (for the warm regions site-I & site-II), and AMPYHS1P and AMPYHS2P (for the cold regions site-I & site-II) were represented. All the individual retrieve information of each sample were also shown in Supplementary Table 1,2,3 & 4, Supplementary Excel Sheet 1,2 & 3.

## 5 Results

### 5.1 Physical characteristics

In the hot regions of both sample locations, variations of temperature were about 10 ° Celsius were seen. There are not much variances in the warmer region since we gathered samples from both sampling points (site-I & II) in the warmer section where the temperature drops and remained at 37° to ensure uniformity in the samples from both locations. On the other hand, the cold region's temperature (stream differs from site-I by 4 ° Celsius) as presented in Table 1. All mud sediment samples from hot, warm, and cold locations fall between 8 and 9.5 on the pH scale as presented in Table 1, suggesting that the sampling sites were alkaline. Due to the near dispersion of each sample location, there is probably not much variance seen with regard to these various natural thermal region.

### 5.2 Diversity indices

**Fig. 2:**
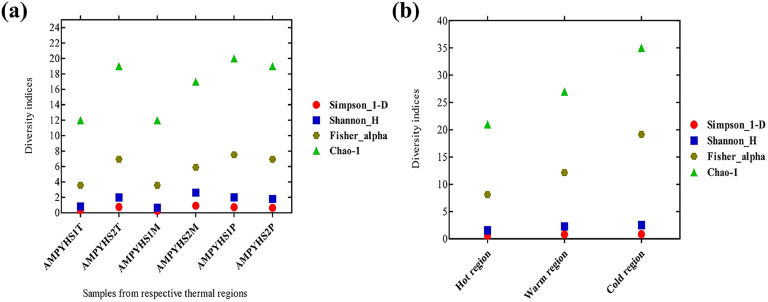
Alpha diversity indices (a) for the corresponding samples; (b) for the corresponding natural temperature regions (hot, warm, and cold).

**Fig. 3:**
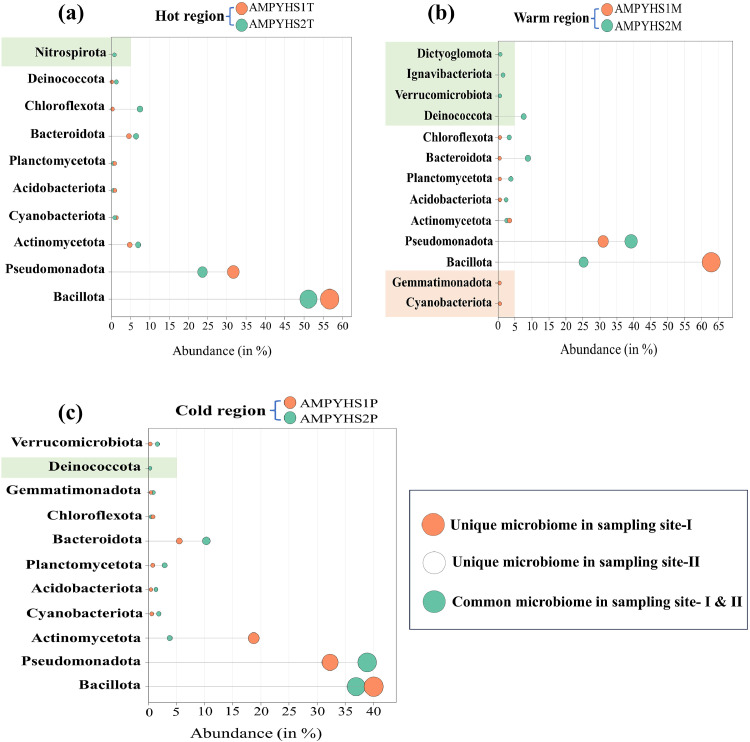
Phylum level abundance of the core microbiome (a) for the corresponding Hot regions (b) for the corresponding Warm regions (c) for the corresponding Cold regions.

**Fig. 4:**
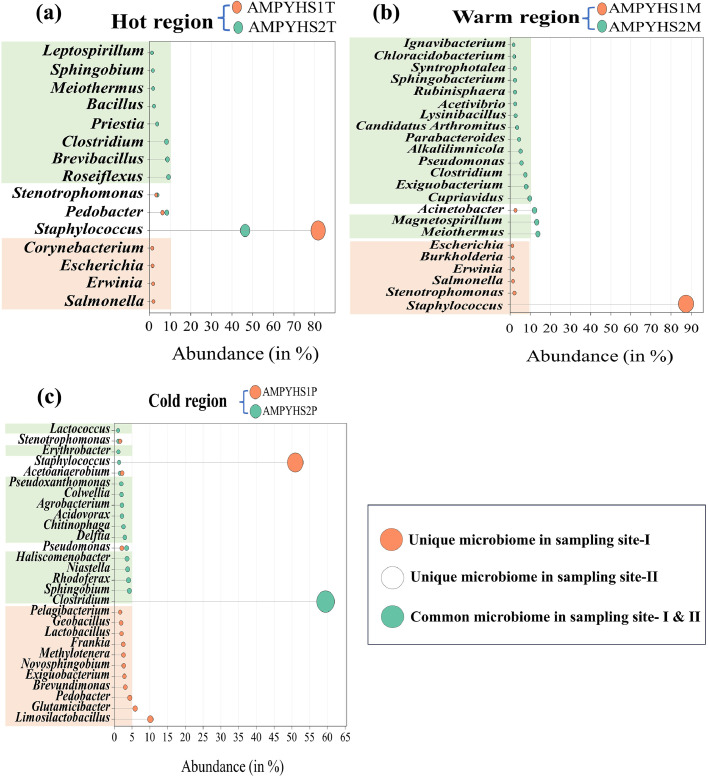
Genus level abundance of the core microbiome (a) for the corresponding Hot regions (b) for the corresponding Warm regions (c) for the corresponding Cold regions.

The analysis revealed that the Fisher alpha diversity index was highest in samples from cold regions (19.14) and lowest in those from hot regions (8.10). A comparison using the Shannon index indicated that cold regions had the highest diversity (2.52), while hot regions exhibited lower diversity (1.57). The Simpson index showed the highest score in warm regions (0.83) and the lowest in hot regions (0.57). The Chao-1 diversity index was also highest in samples from cold regions (35) and lowest in samples from hot regions (21). The alpha diversity analysis, depicted in Fig. 2a and 2b, indicates that taxonomic richness was highest in colder regions and decreased with rising temperatures. The detailed diversity indices are presented in Table 3. The core microbiome was plotted and graphed in Fig. 3a-c and Fig. 4a-c. In Fig. 3a-c, phylum level abundance of the core microbiome is shown whereas Fig. 4a-c shows the genus level abundance of the core microbiome.

**Table 3:** Variation in the genus level of the alpha diversity indices for the corresponding thermal regions.

Sampling sites	Diversity indices			
	Fisher alpha	Shannon_H	Simpson_1-D	Chao-1
Hot region	8.107	1.572	0.5774	21
Warm region	12.15	2.285	0.786	27
Cold region	19.14	2.523	0.8344	35

### 5.3 Phylum level bacterial diversity

**Fig. 5:**
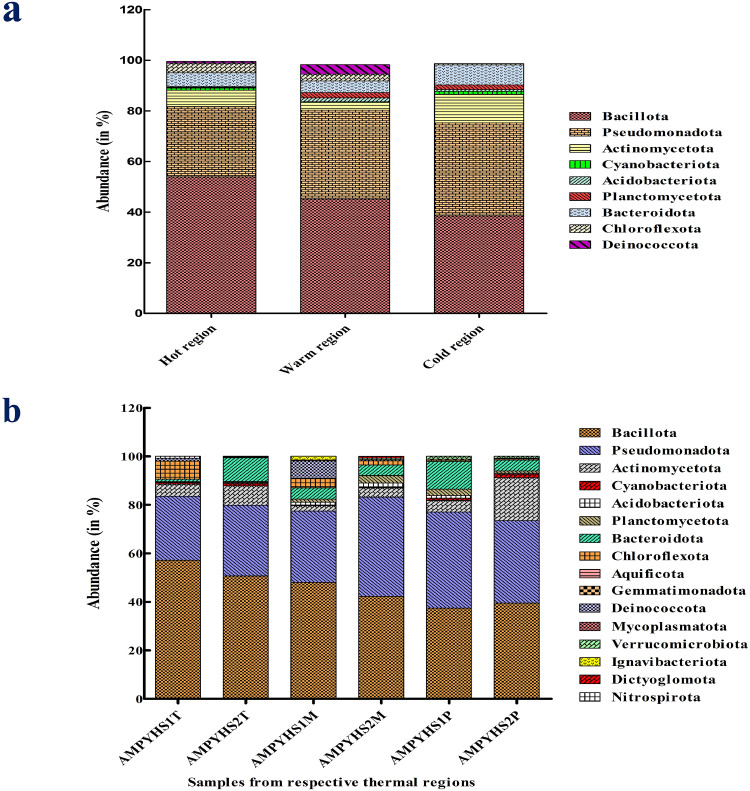
(a) Phylum-level bacterial diversity and corresponding temperature region (b) Depicting the bacterial phylum level diversity at the relevant samples.

The phylum-level richness of bacterial diversity showed that warmer regions had a more diverse population of bacteria than both hot and cold regions Fig. 5a, Supplementary Fig. 2. Certain phyla, such as Bacillota and Chloroflexota, were observed to significantly increased as the temperature rose. Nonetheless, a few phyla, such as Pseudomonadota, Deinococcota, and Verrucomicrobiota, declined as the temperature rose. Besides, all three of these thermal regions (hot, warm and cold) contain a large number of common phyla that are not significantly impacted by temperature variations, including Actinomycetota, Cyanobacteriota, and Bacteroidota. Certain phyla, such as Dictyoglomota and Ignavibacteriota, are exclusively detected in warm region. In contrast, Nitrospirota are exclusively found in hot region only. The hot regions harbour Bacillota as a predominant phylum (53.96 %), followed by Pseudomonadota (27.65 %), Actinomycetota (5.82 %), Bacteroidota (5.46 %), and Chloroflexota (3.83 %). In the warmer regions, the Bacillota was 44.07 % followed by Pseudomonadota (35.15 %), Bacteroidota (4.55 %), Deinococcota (3.81 %), Actinomycetota (3.03 %), Planctomycetota (2.12 %), Chloroflexota (1.80 %) and Acidobacteriota (1.44 %). Whereas, for cold regions, the dominant phyla were Bacillota (38.52 %) followed by Pseudomonadota (35.63 %), Actinomycetota (11.26 %), Bacteroidota (7.90 %), Planctomycetota (1.80 %) and Cyanobacteriota (1.21 %) Fig. 5b, Supplementary Excel sheet 1. Over all the temperatures showed the effect on the colonization of bacterial phyla in each thermal region.

### 5.4 Correlation between abundant phyla, pH and temperature

**Fig. 6:**
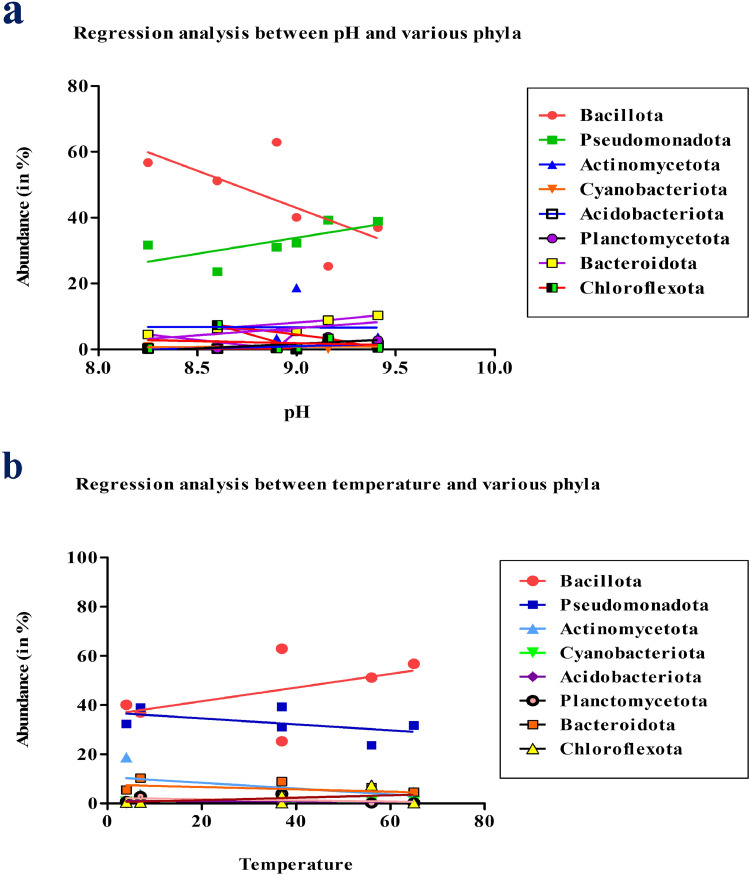
Analysis of a linear regression plot (a) Relationship between pH and several prevalent bacterial phyla (b) Relationship between temperature and several prevalent bacterial phyla.

Regression plot analysis was performed to check the impact of physical parameters like pH and temperature on bacterial diversity in each natural thermal region (hot, warm and cold). Some of the abundant phyla like Bacillota, Pseudomonadota, Actinomycetota, Cyanobacteriota, Acidobacteriota, Planctomycetota, Bacteroidota and Chloroflexota were used for correlation with pH and temperature. The result has shown that some phyla such as Planctomycetota and Pseudomonadota show moderate to high correlations with pH as shown in Fig 6a, none of the p-values indicate statistical significance at the conventional 0.05 threshold. This suggests that there is no strong evidence to conclude that pH significantly affects the abundance of these bacterial phyla. The temperature relationships among the used phyla were examined, and the results showed that the coefficients of determination (r² values) to be 0.9249 for Bacillota, 0.6939 for Pseudomonadota, 0.5391 for Actinomycetota, 0.2244 for Cyanobacteriota, 0.06854 for Acidobacteriota, 0.4893 for Planctomycetota, 0.6127 for Bacteroidota, and 0.9422 for Chloroflexota. These r² values indicate that Bacillota and Chloroflexota have strong correlations, Pseudomonadota, Actinomycetota, Planctomycetota, and Bacteroidota have moderate correlations, while Cyanobacteriota and Acidobacteriota have weak correlations with temperature as shown in Fig 6b. A linear regression plot was performed with genera level bacterial diversity in relation to temperature as shown in Supplementary Fig. 3a and in relation to pH were shown in Supplementary Fig. 3b. The results showed that *Clostridium* had a strong correlation with pH and an inverse correlation with increasing temperature. In contrast, *Staphylococcus* was inversely correlated with increasing pH. We also conducted a linear regression analysis with the OTUs we obtained from phyla, genera and species level, revealing that moderate temperatures had a strong correlation as shown in Supplementary Fig. 4a,b,c. This suggests that temperature variations significantly influence the distribution or abundance of OTUs, emphasizing the importance of moderate temperature conditions in shaping microbial communities.

### 5.5 Genus level bacterial diversity

**Fig. 7:**
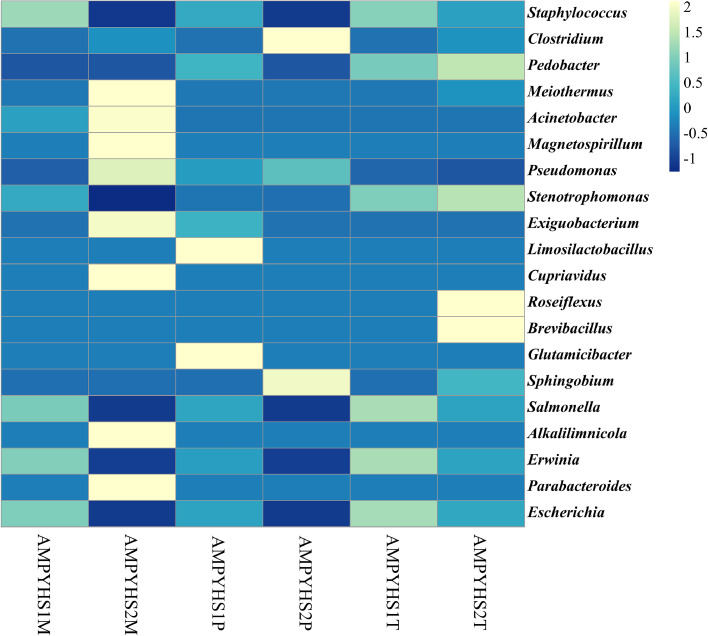
Heat map displaying the diversity of bacteria at the genus level at various temperature regions.

In practically every location, there are dominant genera and distinct bacterial phyla. *Staphylococcus* accounted for 63.96 % of the total population and was the most common genera in the samples from the hot regions, followed by *Pedobacter* (7.27 %), *Roseiflexus* (4.57 %), *Brevibacillus* (4.29 %), *Clostridium* (4.10 %), and *Stenotrophomonas* (3.48 %) were the most common genera. Similarly, the *Staphylococcus* found to be dominated in warmer regions with an abundance of 43.59 %, followed by *Acinetobacter* (7.45 %), *Meiothermus* (6.92 %), *Magnetospirillum* (6.63 %) and *Cupriavidus* (4.89 %) respectively. Cold regions were predominated by *Clostridium* (29.74 %) followed by *Staphylococcus* (26.15 %) and *Limosilactobacillus* (5.06 %). The overall distribution of the microbial population at the genus level is shown in Fig. 7, Supplementary Fig. 5, Supplementary Excel Sheet 2.

### 5.6 Statistical analysis

**Fig. 8:**
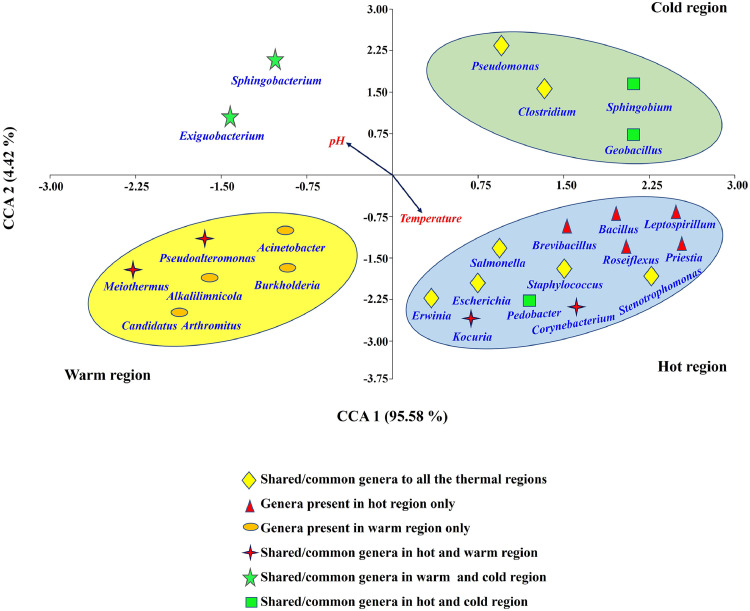
Plots of the Canonical Correspondence Analysis (CCA) for various temperature region.

Temperature, pH, and genus-abundance were the three sets of variables whose relationships were determined using Canonical Correspondence Analysis (CCA). In the gradient thermal regions (hot, warm, and cold), the temperature exhibited both positive and negative relationships with different genera. Different groups were generated using the CCA plot cluster based on different temperatures, pH and genera. Temperature displayed a negative correlation with *Sphingobacterium* and *Exiguobacterium* and a positive correlation with hot followed by warm regions. While two genera—*Sphingobacterium* and *Exiguobacterium*—exhibited a positive correlation with pH, other genera from cold regions showed neither a positive nor negative correlation with pH. The association between pH and genus was found to have limted significance. This clarifies that bacterial colonies favor a specific temperature range for their development. Furthermore, some of genera are shared across all the thermal regions as shown in Fig 8. This might be because the water mixed as they flowed through the hot spring, from the warm region to the frigid region where they ended up.

## 6 Discussion

Soil or mud sediment serves as a reservoir for microbial deposition and has a diverse spectrum of microorganisms both culturable and non-culturable (Malham et al. 2014; Naik et al. 2018). The composition of sediment and its physio-chemical characteristics, such as temperature, pH, environment, nutrient availability, etc., all play a major role in contributing to the microbial diversity of mud sediment (Rousk et al. 2010; Sharp et al. 2014; Chu 2018; Power et al. 2018). Several studies have demonstrated that microbial diversity varies at higher altitudes (Yergeau et al. 2007) in extremely constrained geographical regions (Cho and Tiedje 2000; Green and Bohannan 2006; Martiny et al. 2006). Colman et al. (2016) studied microbial communities in sediments and plankton from 15 Yellowstone National Park springs, with temperatures between 42.5 °C and 90.7 °C and pH ranging from 1.8 to 7.7. The study found that microbes adapted to specific niches, with sediment microbes using mineral-based energy and planktonic ones relying on dissolved oxidants like oxygen. Temperature gradients shape microbial diversity, influencing energy conservation strategies. As temperatures rise, thermophilic microbes dominate, while lower temperatures favour oxygen-dependent metabolisms, demonstrating how environmental factors drive microbial niche differentiation (Colman et al. 2016). The study by Guo et al. (2021) on geothermal fields in Rehai and Banglazhang, in the Tengchong-Longling region of southwestern Yunnan, China and the study of thermal gradients in high-altitude hot springs in North Sikkim both highlight the intricate relationship between environmental factors and microbial community structures in geothermal ecosystems. Guo et al. (2021) focused on how pH, temperature, and sulfate levels shape microbial diversity in hot springs with differing geochemical properties. They found that alkaline, high-temperature springs were primarily influenced by pH and temperature, while acidic, moderate-temperature springs were shaped by sulfate concentrations (Guo et al. 2021).

It is generally believed that the macroclimate, or large-scale variables, influences biodiversity differently than the microclimate, or local conditions, resource availability, etc. (Cockell et al. 2015; Bässler et al. 2016). Very limited study has been done on how various microclimates affect the bacterial diversity in relation to significant temperature gradients. The main goal of the current study was to assess how the three microenvironments with major gradient temperature distributions—from cold to warm to hot, or vice versa—changed the bacterial diversity. The high altitude Indian Himalayan Geothermal Belt was selected as the study's sample area because it contains three different microenvironments coexisting in the same location due to the region's geological topography. The Yumesamdong hot spring provides a unique opportunity to investigate the microbial community structure thriving at variable temperature gradients at close proximity (Kumar et al. 2023b). Thus, the research area offered three different micro-environment regions that have currently been studied: the hot spring (hot region), the path in between hot and cold region (warm region), and the river basin (cold region), as shown in Fig. 1. Three key issues have been addressed here: a) which bacteria live in these settings, how many are dispersed over these areas, and how is the population of their community changing or shrinking in response to gradient temperature b) which is the primary abiotic factors (pH and temperature) influencing the variety of bacteria in these microenvironments.

The results of this investigation showed that both Bacillota and Pseudomonadota bacteria were the predominant phyla. According to research by Chan et al. (2017) on hot-spring mud sediment samples from Malaysian hot springs, Bacillota was the leading and Pseudomonadota was the second most common phylum under temperatures ranges 50–110 °C. The present study's findings are in line with research on the bacterial diversity of several Indian hot springs (Hedlund et al. 2012; Singh and Subudhi 2016; Ghilamicael et al. 2017; Saxena et al. 2017; Sharma et al. 2017; N 2020; Rupasinghe et al. 2022; Das et al. 2021, 2023). Interestingly, the phylum abundances exhibited different patterns with temperature variations. As the temperature fell, Bacillota and Chloroflexota gradually decreased. The fact that this tendency continued everywhere indicates that they mostly prefer hotter climates and that their populations decrease with decreasing temperatures. Conversely, Pseudomonotonadata and Verrucomicrobiota had the opposite tendency, increasing in quantity as the temperature decreased. This demonstrates that in colder environments, Pseudomonadota and Verrucomicrobiota populations grow and thrive. Nitrospirota were exclusively revealed in hot, and warm regions, indicating a limited distribution to this specific habitat. Similarly, Ignavibacteriota and Dictyoglomota were only found in warm region, showing a preference for moderate temperatures and a restricted distribution to these habitats. The frequent distribution and transcriptional activity of Comammox Nitrospira in high temperature habitats are further supported by a study conducted by Zang et al. (2023). The activity of hot spring comammox Nitrospira was further validated by the transcription of genes linked to heat adaptation, which also provided insight into their possible survival strategies (Zhang et al. 2023). In reaction to external stress, cells express heat shock proteins, or HSPs. By creating temperature-dependent complexes, they may also operate as a protective factor for other proteins in addition to their roles in protein assembly, secretion, transcription factor control, and the removal and refolding of damaged proteins (Feng et al. 2007). A moderately thermophilic species of *Ignavibacterium album* was first identified by Lino et al. (2010) from microbial mats in hot spring water sources in Yumata, Nagano, Japan (Iino et al. 2010). Ignavibacteria are known to be thermophilic or thermotolerant, and they are usually found in hot springs with high sulfide concentrations (Podosokorskaya et al. 2024). This suggests that Ignavibacteria can survive in temperatures that are both hot and somewhat warm. Likewise, Dictyoglomota are classified as heterotrophic thermophilic bacteria with a variety of carbon source preferences, which might account for their distribution in warm climates (Leng et al. 2023). In these extremely high-altitude locations, we also found the lowest abundances of Cyanobacteriota, Deinococcota, Nitrospirae, Acidobacteriota, Planctomycetota, and Gemmatimonadetes. Similar findings were also seen in hot spring mud sediment samples from Eritrea's eastern lowlands (Ghilamicael et al. 2017). Another investigation revealed that the two key contributing populations in hot springs with lower temperatures were Cyanobacteriota and Chloroflexota (Nishiyama et al. 2013; Badhai et al. 2015; Ghilamicael et al. 2017; Subudhi et al. 2018; Sharma et al. 2020).

The diversity of bacteria observed at the genus level suggested that distinct bacterial genera were present under a range of gradient temperature conditions. Our results showed that 63.97 % of the total bacterial population in the hot region belonged to the genus *Staphylococcus*, which was the most prevalent type of bacteria there. Followed by *Brevibacillus* (4.29 %), *Clostridium* (4.10 %), *Roseiflexus* (4.57 %), and *Pedobacter* (7.27 %) were present. With *Meiothermus* having the lowest representation at 0.84 %, other genera including *Stenotrophomonas, Priestia* and others were less common, ranging from 3.48 % to 1.06 %. These differences are mostly explained by the different adaptability and thermotolerance of these microorganisms. For instance, *Staphylococcus* is able to withstand high temperatures because of its heat-stable enzymes and heat-shock proteins. Because of geotectonic activity, hot springs—natural geothermal reservoirs with substantial social value—are widely dispersed throughout the Himalayan area and have long been associated with health and spiritual advantages (Wangchuk et al. 2020; Das and Thakur, 2021). Numerous illnesses are treated using balneotherapy, spa therapy, and taking a soak in these springs. However, these springs also contain microbial pathogens with antibiotic and biocide resistant genes (Najar et al. 2020a; Najar et al. 2022a; Sharma et al., 2020, 2022).

In terms of bacterial colonization, the warm region exhibits high abundance compared to cold and hot regions. *Staphylococcus, Acinetobacter, Meiothermus, Magnetospirillum, Cupriavidus, Exiguobacterium, Clostridium, Pseudomonas, Alkalilimnicola*, and *Parabacteroides* were the most prevalent bacterial taxa found in the warm regions. *Meiothermus* and *Exiguobacterium* are thermophiles and psychrophiles, respectively, and their existence indicates that these bacteria has adapted to warmer region besides their optimal growth conditions. This suggests that these bacteria have evolved to the warm region since there are less nutrients availability in the hot and cold regions. Previous research has also demonstrated that *Meiothermus* have been isolated from a variety of hot spring habitats and are moderately thermophilic in nature (Lefevre et al. 2010; Mori et al. 2012; Sheu et al. 2012). A similar study by Podar et al. (2020) found that a wide variety of mesophilic and moderately thermophilic bacteria dominated the 38 °C hot springs in Yellowstone and Iceland (Podar et al. 2020). This finding was further correlated with the higher taxonomic richness in hot springs with lower temperatures (38 °C) compared to thermal areas (50–80 °C).

The most prevalent bacterial taxa retrieved from cold regions were *Clostridium, Staphylococcus, Limosilactobacillus, Glutamicibacter, Pseudomonas, Pedobacter*, and *Sphingobium*. These taxa contributed significantly to the bacterial communities. In addition to psychrophiles and mesophiles, the genus *Geobacillus* is also present in these cold regions. One primary reason for their presence is the water from hot springs, which might carry *Geobacillus* spores downstream, as *Geobacillus* is a spore-forming bacteria.

Some studies shown that the taxonomic richness varies with respect to ambient temperature gradient (Bendia et al. 2018; Podar et al. 2020). The increasing environmental temperature accounts for the decrease in taxonomic richness and diversity of the resident microbial community (Badhai et al. 2015; Choure et al. 2021; Rao et al. 2021). Our results correlate with the study of Podar et al. (2020) and Debnath et al. (2023), where analysis of mud sediments from geothermal hot springs reveals a significant reduction in bacterial diversity with an increase in temperature (Podar et al. 2020; Debnath et al. 2023). The evenness (Shannon) and abundance (Simpson) index of the warm thermal region were the highest (2.28, 0.78) as compared to the hot region (1.57, 0.57), suggesting a higher microbial diversity in the warm gradient. Similarly, an investigation of microbial diversity of hot spring mud sediment samples from two alkaline hot springs Jakrem and Yumthang revealed that the Shannon index was relatively higher for Yumthang (39 °C) than Jakrem (46 °C). Thus, it is possible to hypothesize that there is still a clear correlation between microbial diversity and the hot spring sample's temperature, indicating that temperature is still a key factor in determining the composition of microbial communities. The Chao-1 diversity indices were found to be the highest in warm region samples and lowest in the samples from hot regions. In thermophilic microbial communities, there is a negative correlation between the alpha-diversity indices (Shannon and Chao1) and rising temperatures (Rupasinghe et al. 2022). This association is also supported by a number of studies on the geothermal hot spring habitats (Rupasinghe et al. 2022; Debnath et al. 2023). Numerous study has documented the impact of temperature on the composition of the microbial population in hot springs (Hou et al. 2013; Delgado-Serrano et al. 2014; Badhai et al., 2015, Uribe-Lorío et al., 2019; Dixit et al. 2021; Toshchakov et al. 2021), Many studies on hot spring settings have focused on various environmental factors that influenced the colonization of bacterial communities in a precise thermal region (Rousk et al. 2010; Cole et al. 2013; Wang et al. 2013; Cuecas et al. 2014; Sharp et al. 2014; Najar et al. 2018; Power et al. 2018; Kumar et al. 2023a, b; Shang et al. 2024; Najar et al. 2020a, b; Das et al. 2021a, b).

The CCA plot analysis showed that the abundance of thermophilic bacterial genera such as *Leptospirillum, Brevibacillus, Bacillus, Roseiflexus*, and *Priestia* was strongly correlated with temperature and negatively correlated with pH. Their temperature response aligns with their physiological adaptations, indicating temperature is a major factor influencing microbial community structure across hot, warm, and cold regions. Higher temperatures favor thermophiles, while mesophiles and psychrophiles decrease in richness as temperature increases. This finding is consistent with Guo and Wang's (2012) study in Tengchong, China, which showed temperature significantly determines microbial community composition across a gradient of 55.1 to 93.6 °C (Guo and Wang 2012). Numerous studies (Wang et al. 2013; Mateos-Rivera et al. 2016; Najar et al. 2018; Poddar and Das 2018; Podar et al. 2020; Ruhl et al. 2022) have demonstrated the relationship between temperature and bacterial diversity, but this is the first time that a large temperature range has been covered by high-throughput amplicon sequencing, resulting in unique microenvironments in Yusesamdong, North Sikkim, India. According to a study by Podar et al. (2020), the most important factor influencing bacterial diversity in Iceland's hot springs was temperature. Temperature was shown to have an inverse relationship (p-values of 0.006 and 0.000, respectively) with both phylogenetic diversity and the number of discovered taxa Amplicon sequence variants (ASVs) (Podar et al. 2020). Significant relationships between temperature, diversity indices, and species richness have been found in research; for neutral-alkaline springs like Humboldt's spa, r2 values might reach 0.62. According to Sharp et al. (2014), diversity decreased at very high temperatures and peaked at 24 °C (Sharp et al. 2014). Our findings also demonstrate that more diversified bacterial populations are supported in warmer climates. Many research (Cole et al. 2013; Cuecas et al. 2014; Sharp et al. 2014; Power et al. 2018) certify to the importance of temperature in determining bacterial diversity. pH has an impact on microbial diversity as well (Rousk et al. 2010; Sharp et al. 2014; Power et al. 2018). However, our investigation found a weak relationship between pH and bacterial diversity. Pseudomonadota and pH, on the other hand, showed a minor positive association, which is probably because the settings we analyzed had a restricted pH range of 8.5–9.1.

## 7 Conclusion

This study explored bacterial diversity in the thermal gradient of Yumesamdong, North Sikkim, India, focusing on three closely located region: hot, warm, and cold. The slightly elevated hot region allows hot spring water to flow into the warm area before mixing with a glacial stream. High-throughput amplicon sequencing revealed notable differences in bacterial community composition, with the warm region showing the highest taxonomic richness. We found a weak correlation between bacterial diversity and pH, while temperature emerged as the primary factor shaping community structure. Particularly, Bacillota was the most abundant phylum, followed by Pseudomonadota and Actinomycetota. This study offers valuable insights into microbial dynamics in high-altitude geothermal systems, enhancing our understanding of bacterial adaptation to extreme thermal conditions. The findings may also guide future studies in gradient or contaminated environments, emphasizing the ecological significance of temperature in shaping microbial communities.

## CRediT authorship contribution statement

Nagendra Thakur: Conceptualization, and design of the study, assisted in sample collection, reviewed the draft, and final approval of the version to be submitted. Santosh Kumar: Collected the samples, acquisition of data, analysis and interpretation of data and drafting the article. Sayak Das: Collected the samples, drafting the article, and reviewed the final version. Avinash Sharma and Namrata Jiya: Conducted amplicon sequencing, performed data analysis, and assisted the manuscript writing process. Chirantan Saha, Prayatna Sharma, and Sonia Tamang: Assisted with sample collection, assisted the manuscript writing process and reviewed the draft.

## Declaration of competing interest

The authors declare that they have no known competing financial interests or personal relationships that could have appeared to influence the work reported in this paper.

## Data Availability

No data was used for the research described in the article.

## Appendix Supplementary materials

Image, application 1

Image, application 2

Image, application 3
