# Supplementary material for: Bacterial diversity along the geothermal gradients: insights from the high-altitude Himalayan hot spring habitats of Sikkim
Source: Curr Res Microb Sci. 2024 Nov 7;7:100310. doi: 10.1016/j.crmicr.2024.100310 (PMC11613191; doi:10.1016/j.crmicr.2024.100310)
Supplement: Supplementary file 2 [file mmc2.docx]

**Supplementary Table 1** Phylum level abundance (OTUs) at respective thermal region**s**

| **Sampling Sites** | **Hot region** | | **Warm region** | | **Cold region** | |
| --- | --- | --- | --- | --- | --- | --- |
|  | **Total OTUs** | **Percentage (%)** | **Total OTUs** | **Percentage (%)** | **Total OTUs** | **Percentage (%)** |
| Old Yumesamdong (Site-I) | 291142 | 31.24354 | 348896 | 37.44134 | 291809 | 31.31512 |
| New Yumesamdong (Site-II) | 270167 | 31.52054 | 295397 | 34.46414 | 291550 | 34.01531 |

**Supplementary Table 2** Genus level abundance (OTUs) at respective thermal regions

| **Sampling Sites** | **Hot region** | | **Warm region** | | **Cold region** | |
| --- | --- | --- | --- | --- | --- | --- |
|  | **Total OTUs** | **Percentage (%)** | **Total OTUs** | **Percentage (%)** | **Total OTUs** | **Percentage (%)** |
| Old Yumesamdong (Site-I) | 189668 | 33.92787 | 240555 | 43.03055 | 128810 | 23.04157 |
| New Yumesamdong (Site-II) | 175868 | 38.7675 | 144357 | 31.82137 | 133423 | 29.41113 |

**Supplementary Table 3** Species level abundance (OTUs) at respective thermal regions

| **Sampling Sites** | **Hot region** | | **Warm region** | | **Cold region** | |
| --- | --- | --- | --- | --- | --- | --- |
|  | **Total OTUs** | **Percentage (%)** | **Total OTUs** | **Percentage (%)** | **Total OTUs** | **Percentage (%)** |
| Old Yumesamdong (Site-I) | 53473 | 30.79994 | 54841 | 31.58789 | 65300 | 37.61217 |
| New Yumesamdong (Site-II) | 94423 | 29.23041 | 131840 | 40.81355 | 96767 | 29.95604 |

**Supplementary Table 4** Metagenomic samples with Accession numbers

| **SL No.** | **Sample Code** | **Bio Project** | **Bio Sample** | **SRA** |
| --- | --- | --- | --- | --- |
| 1 | NY1M | PRJNA948183 | SAMN33879225 | SRR23952872 |
| 2 | NY2M | PRJNA948345 | SAMN33890424 | SRR23969232 |
| 3 | NY3T | PRJNA948352 | SAMN33900305 | SRR23954778 |
| 4 | NY4T | PRJNA948359 | SAMN33900391 | SRR23954926 |
| 5 | NY5P | PRJNA949106 | SAMN33924774 | SRR23976180 |
| 6 | NY6P | PRJNA949121 | SAMN33924958 | SRR23976394 |
| 7 | Y1M | PRJNA949207 | SAMN33937723 | SRR23980953 |
| 8 | Y2M | PRJNA949222 | SAMN33939807 | SRR23981776 |
| 9 | Y1T | PRJNA949357 | SAMN33942261 | SRR23984900 |
| 10 | Y2T | PRJNA949362 | SAMN33942381 | SRR23984907 |
| 11 | Y1P | PRJNA949289 | SAMN33941960 | SRR23984854 |
| 12 | Y2P | PRJNA949348 | SAMN33942063 | SRR23984856 |
