# Supplementary material for: Bacterial diversity along the geothermal gradients: insights from the high-altitude Himalayan hot spring habitats of Sikkim
Source: Curr Res Microb Sci. 2024 Nov 7;7:100310. doi: 10.1016/j.crmicr.2024.100310 (PMC11613191; doi:10.1016/j.crmicr.2024.100310)

**Supplementary Fig. 1** Solfataric mud sediments collected from the sampling sites of the hot springs.

**
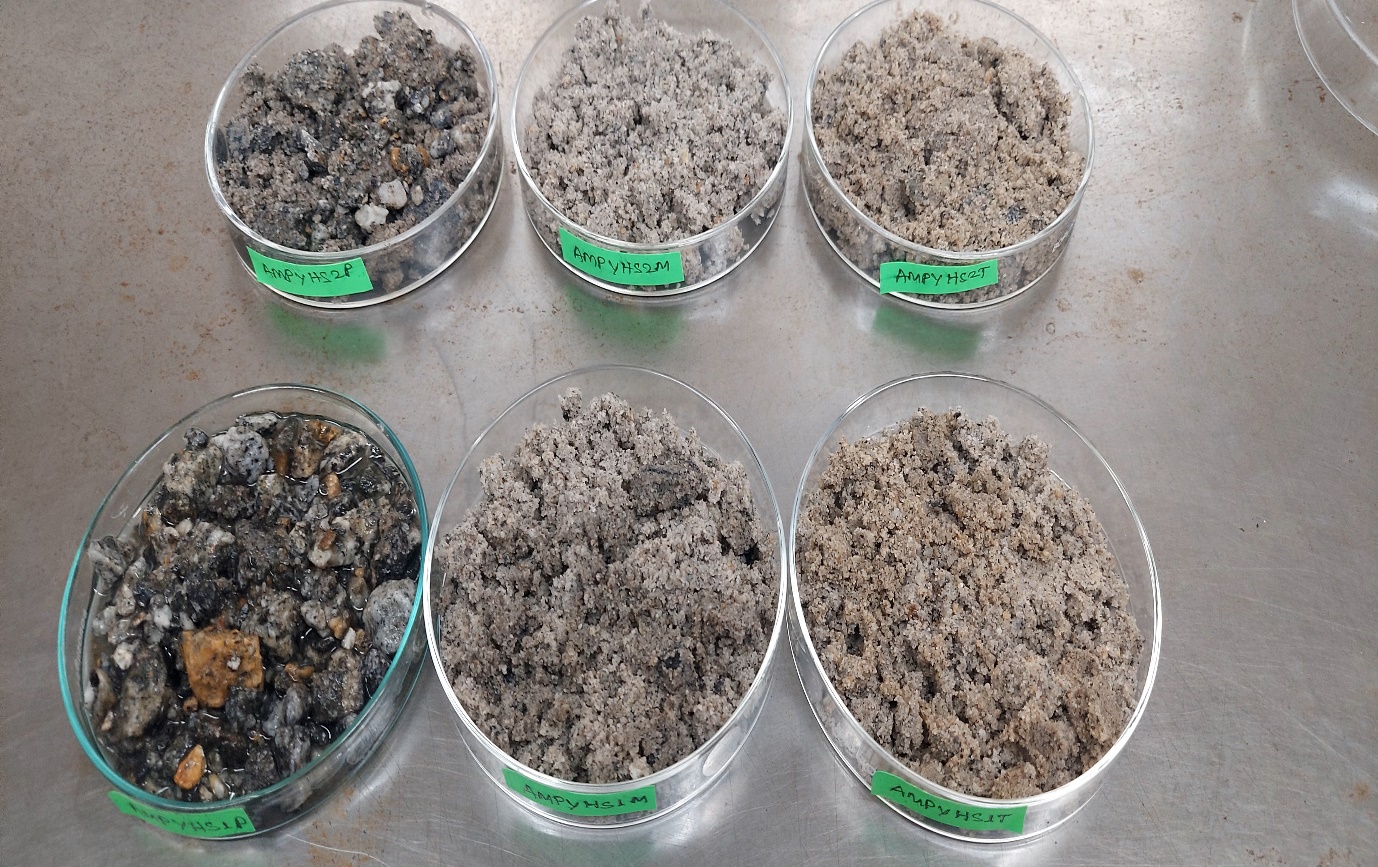
**

**Supplementary Fig. 2** Phylum-level bacterial diversity at the relevant temperate region samples.


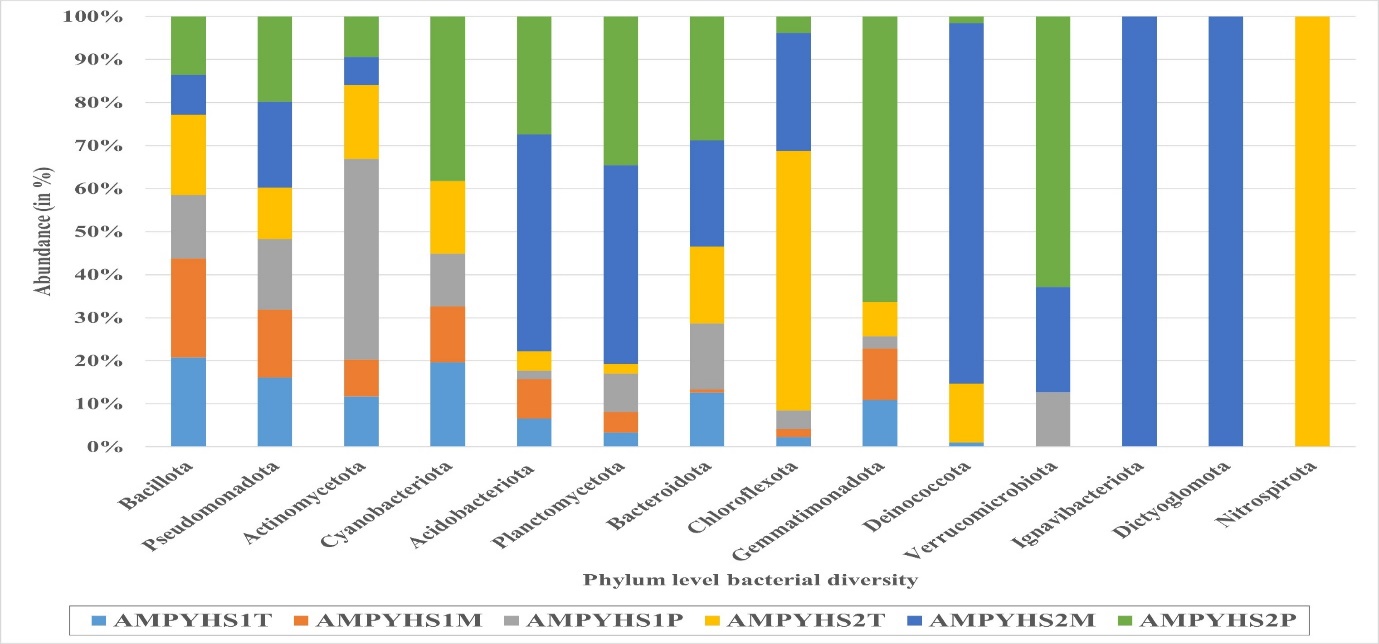


**Supplementary Fig. 3** Analysis of a linear regression plot (a) Relationship between temperature and several prevalent bacterial genera (b) Relationship between pH and several prevalent bacterial genera.


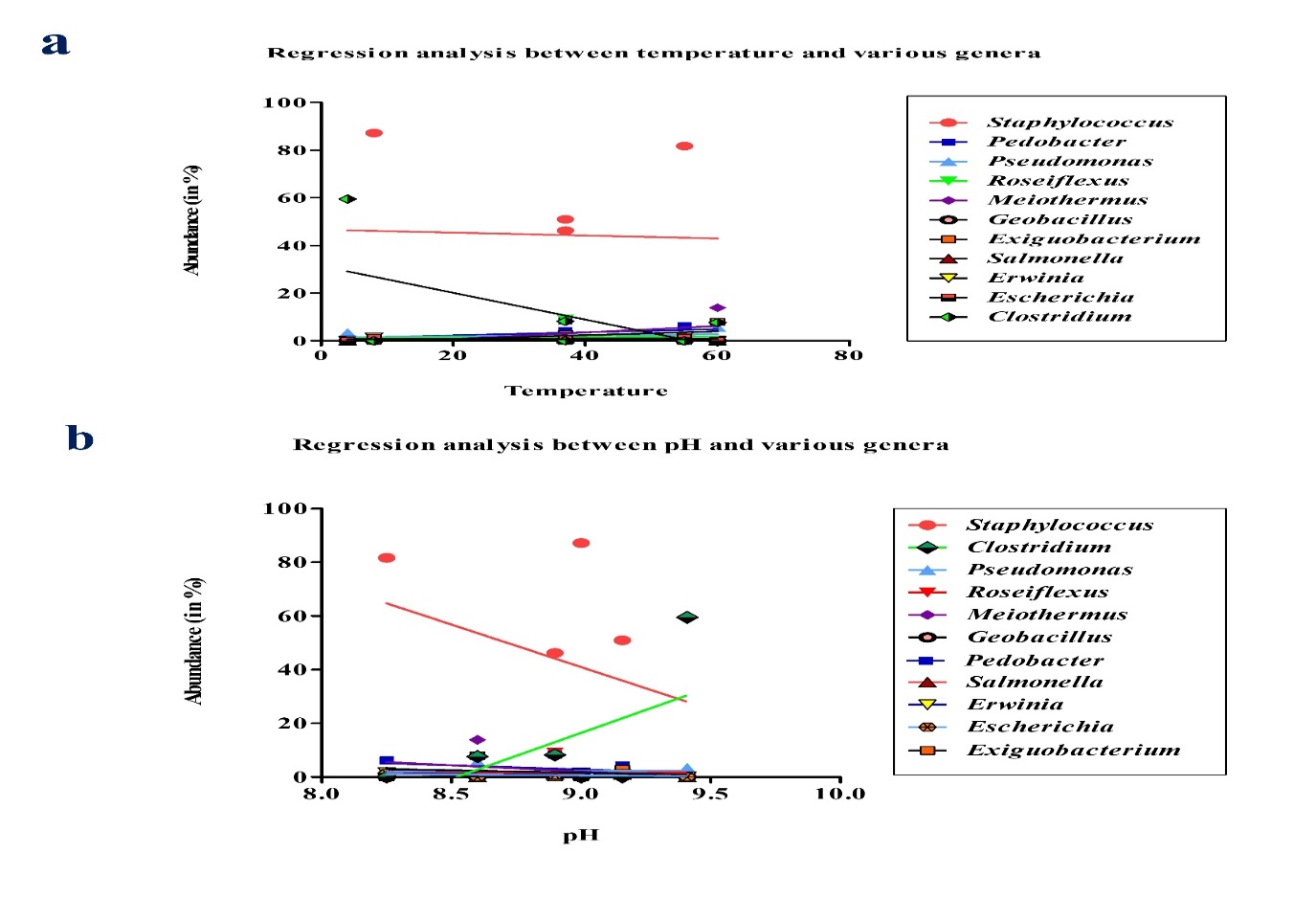


**Supplementary Fig. 4** Analysis of a linear regression plot (a) Relationship between microbial OTUs and prevalent bacterial phyla (b) Relationship between microbial OTUs and prevalent bacterial genera (c) Relationship between microbial OTUs and prevalent bacterial species


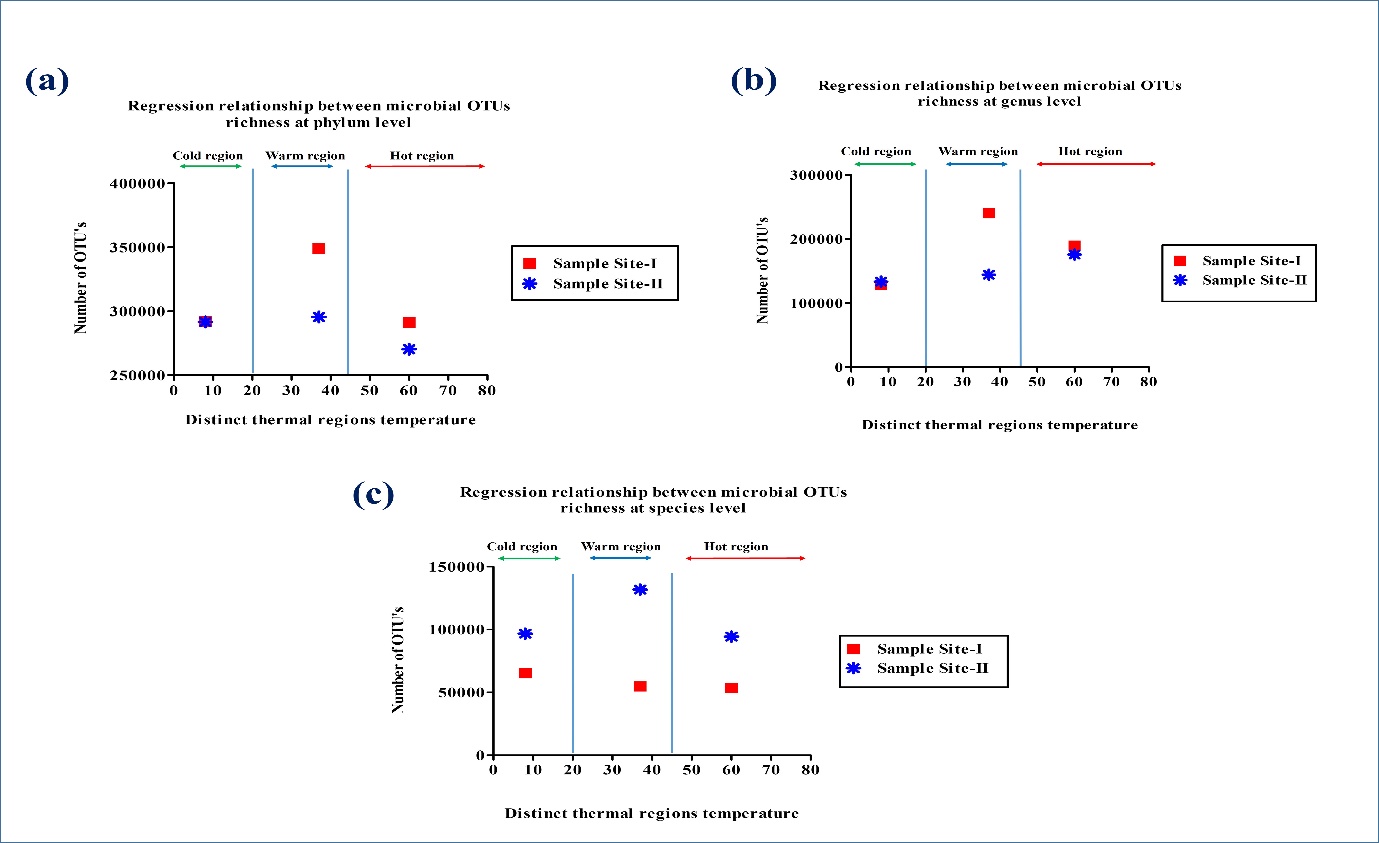


**Supplementary Fig. 5** Genus-level bacterial diversity at the relevant temperate region samples


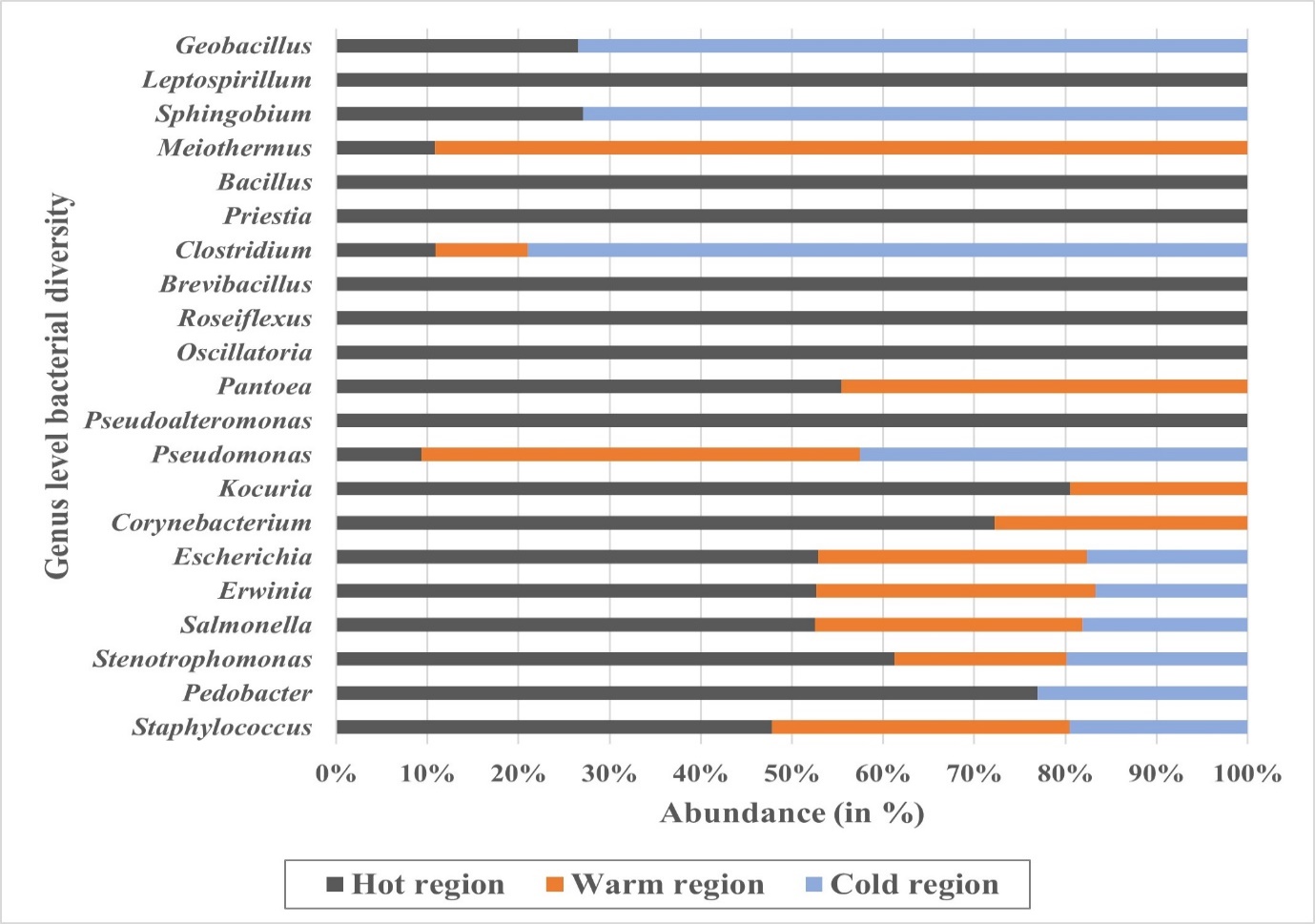

Supplement: Supplementary file 3 [file mmc3.docx]
